# The Dynamic Interplay between HIV-1, SAMHD1, and the Innate Antiviral Response

**DOI:** 10.3389/fimmu.2017.01541

**Published:** 2017-11-10

**Authors:** Jenna M. Antonucci, Corine St. Gelais, Li Wu

**Affiliations:** ^1^Center for Retrovirus Research, Department of Veterinary Biosciences, The Ohio State University, Columbus, OH, United States

**Keywords:** HIV-1, sterile alpha motif and HD-domain containing protein 1, infection, innate immunity, myeloid cells

## Abstract

The innate immune response constitutes the first cellular line of defense against initial HIV-1 infection. Immune cells sense invading virus and trigger signaling cascades that induce antiviral defenses to control or eliminate infection. Professional antigen-presenting cells located in mucosal tissues, including dendritic cells and macrophages, are critical for recognizing HIV-1 at the site of initial exposure. These cells are less permissive to HIV-1 infection compared to activated CD4^+^ T-cells, which is mainly due to host restriction factors that serve an immediate role in controlling the establishment or spread of viral infection. However, HIV-1 can exploit innate immune cells and their cellular factors to avoid detection and clearance by the host immune system. Sterile alpha motif and HD-domain containing protein 1 (SAMHD1) is the mammalian deoxynucleoside triphosphate triphosphohydrolase responsible for regulating intracellular dNTP pools and restricting the replication of HIV-1 in non-dividing myeloid cells and quiescent CD4^+^ T-cells. Here, we review and analyze the latest literature on the antiviral function of SAMHD1, including the mechanism of HIV-1 restriction and the ability of SAMHD1 to regulate the innate immune response to viral infection. We also provide an overview of the dynamic interplay between HIV-1, SAMHD1, and the cell-intrinsic antiviral response to elucidate how SAMHD1 modulates HIV-1 infection in non-dividing immune cells. A more complete understanding of SAMHD1’s role in the innate immune response to HIV-1 infection may help develop stratagems to enhance its antiviral effects and to more efficiently block HIV-1 replication and avoid the pathogenic result of viral infection.

## Introduction

Innate immunity is the cell-intrinsic defense mechanism that senses incoming pathogens and is characterized by type-I interferon (IFN-I) induction and the release of inflammatory cytokines that upregulate antiviral IFN-stimulated genes (ISGs) (1, 2). The activation of the innate response to pathogens is dependent on cellular pattern recognition receptors (PRRs) that detect pathogen-associated molecular patterns (PAMPs), including viral structures or nucleic acids. Interferon-inducible protein IFI16 and cyclic GMP-AMP synthase (cGAS) are cytosolic sensors of HIV-1 that detect viral DNA (3, 4). Recognition of PAMPs results in induction of IFN-I and ISGs to control initial infection and spread, while the concomitant induction of the inflammatory response and cytokines can initiate adaptive immune responses (5, 6). Modulation of IFN-I activation is essential for viral clearance. However, overstimulation of IFN pathways can lead to inflammatory autoimmune disease (7).

HIV-1 is sensitive to ISGs and the IFN-induced antiviral response; so it is not surprising that HIV-1 is a poor inducer of IFN (8). HIV-1 benefits from evading innate immune activation and utilizes a variety of tactics to escape detection (9, 10). Professional antigen-presenting cells located in mucosal tissues, including dendritic cells (DCs) and macrophages, are critical for recognizing HIV-1 at the site of initial exposure. However, these cells are less permissive to HIV-1 infection compared to activated CD4^+^ T-cells, mainly due to host restriction factors that control the establishment or spread of viral infection. Several host proteins can restrict HIV-1 at various points in the viral lifecycle, including APOBEC proteins, TRIM5α, and tetherin (11–13). However, HIV-1 can exploit innate immune cells and their cellular factors to avoid detection and clearance by the host immune system (13).

SAMHD1 is host protein capable of blocking replication of retroviruses and several DNA viruses in cells (14–18). SAMHD1 is constitutively expressed at various levels in all cell types and highly expressed in myeloid lineage and resting CD4^+^ T-cells (14, 15, 19). IFN-I treatment increases SAMHD1 expression in certain cell types with low endogenous SAMHD1 levels (20, 21). SAMHD1 has been implicated as a negative regulator of the IFN-I inflammatory response (22–24), however, the underlying mechanism is not fully understood. While HIV-2 encodes the SAMHD1 antagonist Vpx, the more pathogenic HIV-1 does not. It was hypothesized that HIV-1 lacks a countermeasure against SAMHD1 because it is beneficial for infection. In this review, we will discuss the contributions of SAMHD1 to both the direct restriction of HIV-1 and to the modulation of the antiviral innate response and to analyze the hypothesis that HIV-1 restriction by SAMHD1 leads to a diminished induction of innate immunity.

## Innate Immune Sensing of HIV-1

**Figure 1:**
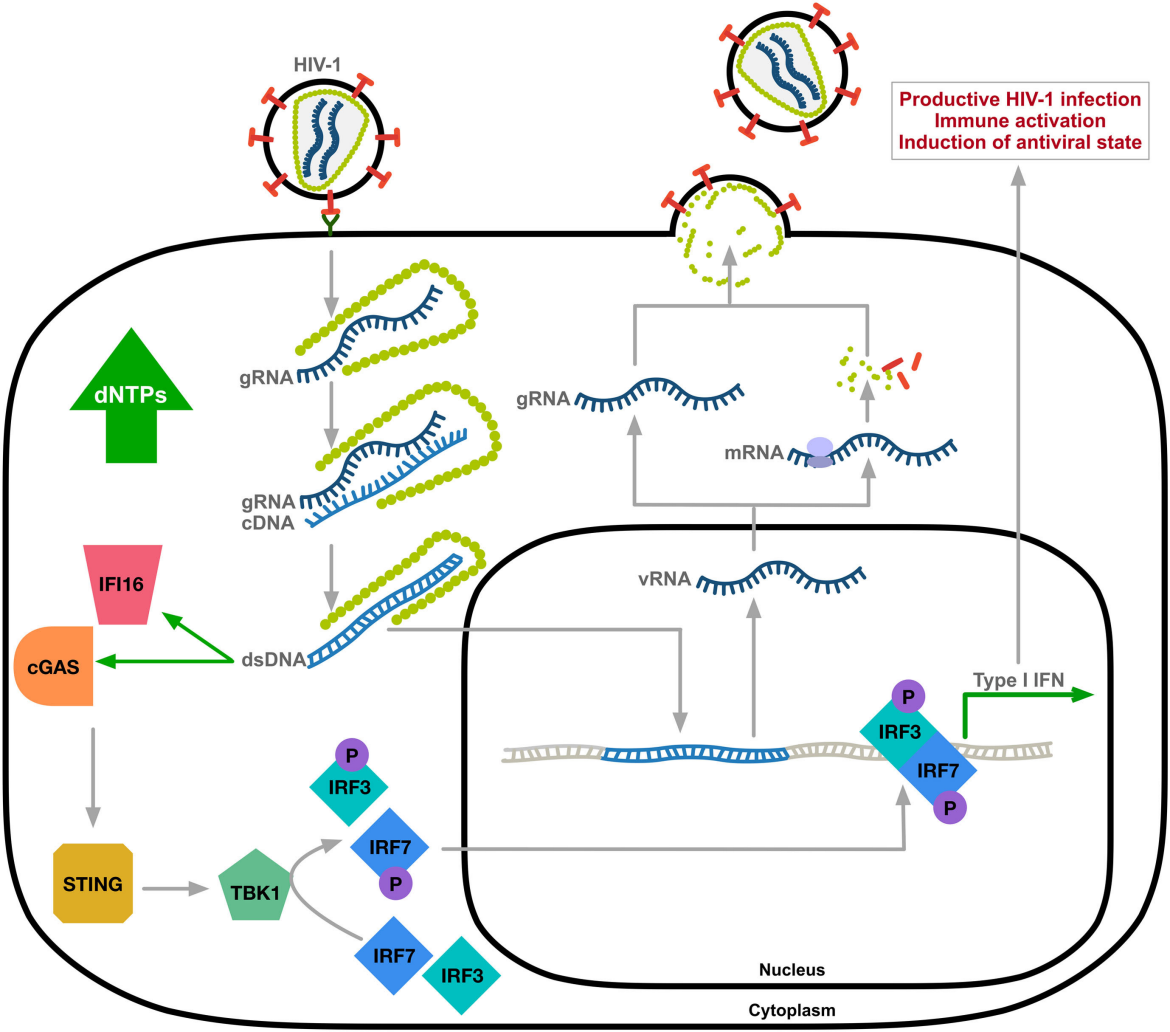
Innate immune sensing of HIV-1 DNA. HIV-1 undergoes uncoating through the interaction between viral capsid and host factors (31, 32). Reverse transcribed HIV-1 DNA, mainly abortive transcripts, activates cytosolic DNA sensors IFI16 and cyclic GMP-AMP synthase (cGAS) resulting in TANK-binding kinase 1 (TBK1)-mediated phosphorylation and nuclear translocation of hetero-or-homo dimers of interferon regulatory factor-3 (IRF3) and IRF7 and induction of type-I IFN response. Expression of ISGs allows for immune activation and the induction of an antiviral state of the cell. gRNA, HIV-1 genomic RNA; cDNA, complementary DNA; vRNA, viral RNA; dsDNA, double-stranded DNA; STING, stimulator of IFN genes; the letter P indicates phosphorylation.

Although HIV-1 can be sensed by the innate immune system, the prevailing theory is that HIV-1 avoids immune surveillance through poor replication in immune cells causing ineffective triggering of innate cytosolic sensors (25). Several studies have identified the molecular basis of cytosolic sensors important for targeting viral pathogens. Here, we focus on HIV-1 DNA as a trigger of the innate antiviral response. After sensing viral DNA, cGAS generates the second messenger, cyclic guanosine monophosphate-adenosine monophosphate (26–28), that activates the stimulator of IFN genes (STING) (6). STING activation leads to phosphorylation of TANK-binding kinase 1 (TBK1) and the subsequent phosphorylation and dimerization of IFN-regulatory transcription factors IRF3 and IRF7. Nuclear translocation of the IRF3/IRF7 homo-or-hetero dimers will activate IFN-I gene expression (Figure 1). This signaling cascade results in an upregulation of IFN-I and ISGs as a defense against viral infection (29, 30). Reverse transcribed HIV-1 DNA was identified as the trigger to the cGAS-STING pathway (3). Although cGAS is the primary sensor of cytosolic viral DNA, IFI16 can also act as a sensor of HIV-1 single-stranded DNA that induces an IFN-β response in macrophages by a cGAS-STING-dependent pathway (4).

## Introduction to SAMHD1

Human SAMHD1 is a 626-amino acid protein containing an N-terminal nuclear localization signal followed by a sterile-alpha motif and histidine/aspartic acid (HD) domain. SAMHD1 is a deoxynucleoside triphosphate triphosphohydrolase (dNTPase) (33, 34) that converts dNTPs into the constituent deoxynucleoside and inorganic triphosphate upon stimulation by dGTP or GTP (33–35). SAMHD1 and ribonuclease reductase, the enzyme responsible for *de novo* dNTP synthesis through the conversion of ribonucleotide diphosphates to deoxyribonucleotides (36), are allosterically regulated to achieve balanced intracellular dNTP levels in a cell-cycle-dependent manner (37). During G_1_ to S-phase transition in actively proliferating cells, ribonuclease reductase expression increases, leading to expansion of the dNTP pool to facilitate DNA synthesis (38, 39). The activity of SAMHD1 is activated by high dNTP levels, and degradation of nucleic acids in the absence of DNA replication protects the cell from innate immune activation and cancer development (40, 41). Mutations in SAMHD1 that affect its enzyme activity are associated with Aicardi-Goutières syndrome (AGS), an encephalopathic autoimmune disease characterized by symptoms mimicking chronic viral infection (22). The accumulation of intracellular dNTPs caused by mutations in the genes encoding proteins involved in nucleic acid metabolism, including SAMHD1 and TREX1 (42), are sensed by PRRs, resulting in aberrant production of IFN-I (43). AGS patients present with increased production of IFN-α, the chemokine most characteristic of congenital virus infection. AGS patients with SAMHD1 mutations can present with signs of lupus erythematosus, with many symptoms mimicking those of HIV-1 infection (22, 44). Furthermore, cells isolated from AGS patients with homozygous *SAMHD1* mutation revealed that SAMHD1-deficient monocytes supported productive infection by HIV-1 (20), suggesting a link between SAMHD1 function in both autoimmunity and HIV-1 restriction.

Long interspersed element 1 (LINE-1) is the only autonomous and active human retroelement capable of producing new genomic insertions through its endogenous endonuclease and reverse transcriptase activities (45, 46). A study on AGS-related SAMHD1 mutations indicate that all disease-related mutations reduced LINE-1 inhibition in dividing cells (47). Recent work suggests that SAMHD1 potently blocks LINE-1 transposition in cycling cells by triggering the sequestration of LINE-1 ORF1p into stress granules (48). Impaired inhibition of LINE-1 retrotransposition may lead to triggering of the autoimmune response by stimulating toll-like receptors (TLRs) (49), although this has not been confirmed. Impaired dNTPase activity and LINE-1 suppression by mutant SAMHD1 could explain the chronic inflammatory response characteristics of AGS disease. These studies outlining the pathogenic effect of SAMHD1 deficiency on autoimmune disease implicate SAMHD1 as a negative regulator of the innate immune system.

## SAMHD1-Mediated HIV-1 Restriction

HIV-1 replicates inefficiently in non-diving cells, such as quiescent CD4^+^ T-cells, DCs, and monocytes. HIV-1 infection can be enhanced in these cells by Vpx, an accessory protein encoded by HIV-2 and certain lineages of simian immunodeficiency viruses (SIVs) (50, 51). This hinted at the existence of a cellular restriction factor counteracted by Vpx (50). SAMHD1 was identified as the mystery HIV-1 restriction factor by a mass spectrometry analysis of cellular proteins immunoprecipitated from cells expressing Vpx (14, 15). Vpx interacts with the C-terminal domain of SAMHD1, thereby initiating proteasomal degradation by an E3 ubiquitin ligase complex, and relieving SAMHD1-mediated lentiviral restriction (14, 15, 52, 53).

**Figure 2:**
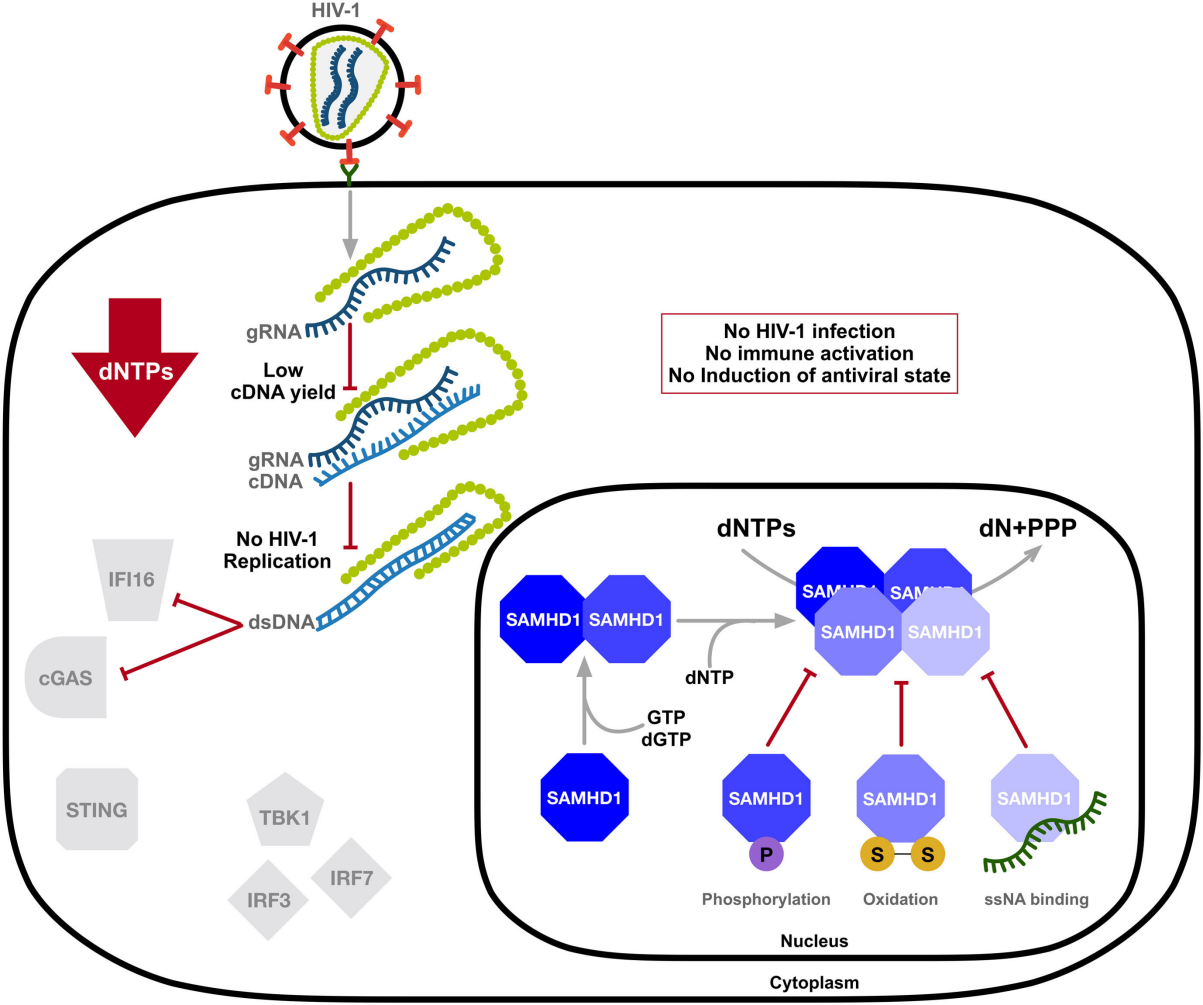
SAMHD1 negatively regulates the innate immune sensing of HIV-1 DNA. SAMHD1 blocks HIV-1 infection through intracellular dNTP depletion, thus preventing the accumulation of viral DNA accessible to sensing by IFI16 and cyclic GMP-AMP synthase (cGAS) and the activation of the type-I interferon (IFN-I) response. The dNTPase activity of SAMHD1 is structurally regulated. Consecutive binding of dGTP/GTP and any dNTP to two allosteric sites provokes formation of the catalytically active tetramer, which can be destabilized by phosphorylation, oxidation, or the binding of single-stranded nucleic acids (ssNAs). dN, deoxynucleosides; PPPs, triphosphate; two linked letters S indicate the disulfide bond.

The mechanism and modulation of SAMHD1-mediated HIV-1 restriction is an area of intense scrutiny (Figure 2). Overexpression of SAMHD1 in PMA-treated monocytic U937 cells results in a depletion of dNTP levels (54). It was later confirmed that SAMHD1 restricts the replication of retroviruses and several DNA viruses by depleting the concentration of intracellular dNTPs to levels insufficient to support viral DNA synthesis (14–18, 54, 55). Structural studies strengthened a model of nucleotide-dependent tetramer assembly of SAMHD1 (56–58), where GTP binds to guanine-specific allosteric sites and dNTP binds to non-specific activator sites, initiating the formation of enzymatically active tetramers with the catalytic core of the HD domain (33, 34, 37, 59). Moreover, binding of single-stranded nucleic acids (ssNAs) to the dimer–dimer interface of SAMHD1 inhibits the formation of the catalytically active tetramer (60).

As SAMHD1 is also highly expressed in activated CD4^+^ T-cells that support productive infection, several studies demonstrated posttranslational modification as a means of mechanistic regulation of SAMHD1 function in restricting HIV-1. SAMHD1 is phosphorylated at several residues; however, phosphorylation of threonine 592 was identified as essential for the negative modulation of its HIV-1 restriction activity (61–65) and tetramer formation (66, 67). SAMHD1 is phosphorylated by cyclin-dependent kinase 1 (CDK1) and CDK2 in complex with cell cycle regulatory protein cyclin A. This regulation of SAMHD1 function is associated with the cell cycle, as CDK1 and cyclin A are highly expressed in dividing cells. Furthermore, S-phase requires elevated dNTP levels, indicating modulation of the dNTPase activity of SAMHD1 during the cell cycle (64). SAMHD1 protein levels may be altered during various stages of the cell cycle depending on different cell types (68, 69). Interestingly, proliferation-induced oxidation of SAMHD1 by hydrogen peroxide reversibly inhibits its dNTPase activity through the formation of tetramer-inhibiting disulfide bonds (70), suggesting a dynamic structure-based regulatory mechanism of SAMHD1’s dNTPase activity that is influenced by the cell cycle (Figure 2).

Although the accepted consensus is that SAMHD1 restricts HIV-1 infection through the depletion of intracellular dNTPs, several studies suggested the existence of an additional yet-undiscovered mechanism of SAMHD1-mediated retroviral restriction. This undefined antiviral activity appears to be dependent on phosphorylation (61, 63, 65) and is not fully dependent on low dNTP levels (71). SAMHD1 acts as a ssNA binding protein that degrades single-stranded DNA and RNA *via* a metal-dependent 3′–5′ exonuclease activity *in vitro* (72–74). It has been suggested that SAMHD1 utilizes its nucleic acid binding potential to exert a ribonuclease activity against incoming HIV-1 genomic RNA in a phosphorylation-dependent manner (75). SAMHD1 was shown to restrict retroviruses though degradation of HIV-1 RNA in human monocyte-derived macrophages (MDMs), monocytes, and CD4^+^ T-cells (75, 76). It was proposed that SAMHD1 degrades incoming HIV-1 gRNA, thereby restricting infection and preventing innate immune sensing of viral nucleic acids. However, recent studies have been unable to confirm the controversial findings (55, 77–79). As a nuclear-localized protein (80), incoming viral genomic RNA would be inaccessible by SAMHD1 for hydrolysis. Additional studies showed that dNTPase inactive SAMHD1 mutant retained exonuclease activities *in vitro*, indicating the exonuclease activity could not be attributed to the known dNTP-binding active site (77). Seamon et al. (77) suggested that the nuclease activity attributed to SAMHD1 was due to contamination during purification. Cell-based assays also failed to recapitulate the findings, thereby confirming the lack of SAMHD1 RNase activity to restrict HIV-1 in infected cells (55, 78). Ryoo et al. suggested that the differences in experimental conditions are responsible for the conflicting results, including a shorter infection time and the use of RNaseH-defective reverse transcriptase (81). They further identified SAMHD1 as a phosphorolytic not hydrolytic ribonuclease (82).

## The Intersection of HIV-1, SAMHD1, and the Innate Antiviral Response

SAMHD1 cDNA was originally identified as a ortholog of the mouse IFN-γ-induced gene *Mg11* in human DCs (83). A link to the innate immune response was strengthened by the discovery that cytokines, including toll-like agonists and IFNs, can induce SAMHD1 expression (84, 85). Cell lines treated with IFN-I (21, 86) and human primary monocytes treated with IFN-α and IFN-γ (20, 84, 87) show enhanced expression of SAMHD1. While SAMHD1 is highly expressed in MDMs, monocyte-derived dendritic cells (MDDCs), and primary CD4^+^ T-cells, IFN treatment does not increase SAMHD1 protein levels further (21, 88–90). However, treatment of MDMs and MDDCs with IFN-I results in reduced phosphorylation of SAMHD1 at residue T592 (61), indicating a shift from catalytically inactive to active SAMHD1. Interestingly, the *SAMHD1* promoter is a direct target of IRF3. The overexpression and activation of IRF3 enhances SAMHD1 promoter activity in HeLa cells (86).

HIV-1 does not trigger a sterilizing immune response (91) and is a poor activator of inflammatory pathways (8), resulting in an impaired response to HIV-1 and the development of persistent infection. The DC response to HIV-1 infection contributes to this dysfunctional immune response (92). Myeloid cells constantly sample the cellular environment to identify pathogens and send out danger signals in the form of IFN-I. DCs are essential for activating the adaptive immune response to infection, as maturation leads to T-cell responses through antigen priming (91, 93). Interestingly, HIV-1 infects DCs without activating an effective antiviral response. As SAMHD1 limits HIV-1 cDNA synthesis in myeloid cells (14, 54), it was hypothesized that degradation of SAMHD1 by Vpx in DCs would result in productive HIV-1 infection and the synthesis of viral proteins that would directly enter antigen presentation, thereby strengthening the T-cell response to infection (94). This could be why the *vpx* gene was lost from the ancestor of HIV-1 during the coevolution of primate SAMHD1 and lentiviruses (95).

Vpx-mediated degradation of SAMHD1 in DCs leads to enhanced HIV-1 infection, and studies in primary MDMs and MDDCs indicate that Vpx-mediated SAMHD1 degradation results in cGAS stimulation and IRF3 activation (3). Early work suggested that enhanced infection by SAMHD1 depletion leads to DC maturation (94). A study utilizing coculture of autologous activated CD4^+^ T lymphocytes with SAMHD1-deficient MDDCs infected with primary clinical HIV-1 isolates indicated enhancement of both infection and IFN response (96). Interestingly, cocultured primary T-lymphocytes, but not HIV-1, trigger a decrease in SAMHD1 expression in MDDCs independent of dNTP levels (96). This study suggests that crosstalk between lymphocytes and DCs induces downregulation of SAMHD1 expression, a requirement for stimulation of HIV-1 production in DCs, thereby inducing the innate sensing of HIV-1 and DC maturation (96).

Conversely, recent work indicates that DC maturation, measured by CD83 and CD86 expression, does not occur in SAMHD1-deficient cells due to additional manipulation of the innate immune system by HIV-1 (97). HIV-1 suppresses TLR-induced maturation of DCs independent of SAMHD1 expression, although Vpx-mediated depletion of SAMHD1 enhanced the effect of HIV-1 infection on lipopolysaccharide-induced DC maturation (97). Vesicular stomatitis virus G-protein-pseudotyped HIV-1 suppressed maturation similar to strains containing HIV-1 envelope protein, suggesting that viral replication, not envelope-receptor interactions, is required for suppression of maturation (97). Removing the SAMHD1-mediated block of reverse transcription resulted in a stronger suppression of maturation. Although infection and subsequent innate immune sensing in DCs is blocked by SAMHD1, HIV-1 maintains an additional SAMHD1-independent mechanism of suppressing DC maturation through downregulation of TLRs (97).

Two additional models suggest that, in MDDCs, HIV-1 attempts to hide its genomic RNA and newly synthesized cDNA from cytosolic sensors by obstructing the nucleic acids using viral capsid. The models differ with respect to the effect of recruitment of cellular cyclophillins and cleavage and polyadenylation-specific factor 6 (CPSF6) by capsid. One model suggests increased cyclophillin A (CypA) binding to the capsid increases sensitivity to innate sensing (94), while another proposes CypA binding coordinates uncoating, reverse transcription, and nuclear import of the preintegration complex (98), all to minimize the exposure of viral nucleic acids to cytosolic sensors. Future work is needed to clarify the contribution of CypA and SAMHD1 to the negative regulation of the innate immune response in myeloid cells to provide insight into HIV-1 mechanisms of evasion.

Non-cycling CD4^+^ T-cells and macrophages are less permissive to HIV-1 because of SAMHD1. However, during HIV-1 infection *in vivo*, activated CD4^+^ T-cells and macrophages are infected due to phosphorylation of SAMHD1. Although cytosolic HIV-1 DNA is abundant in these permissive cells, a cell-autonomous IFN response is not triggered (99). This is due at least in part to host protein TREX1. As a single-stranded DNA exonuclease, TREX-1 digests cytoplasmic DNA from retroviral DNA intermediates, thereby preventing the activation of mislocalized DNA by an innate immune sensor (99). Cytosolic HIV-1 DNA is accumulated in HIV-1 infected TREX1-deficient CD4^+^ T-cells and macrophages, which leads to inhibition of TBK1-dependent IFN-I response (99). This suggests a competition between two DNA sensors: cGAS leading to antiviral effects, and TREX1 leading to enhanced viral replication (100).

## Remaining Questions

Although it is clear that HIV-1 utilizes a variety of mechanisms to evade myeloid cell activation, controversial questions still exist. Conflicting reports could be due to the use of different cell types, and the differential use of clinical HIV-1 isolates, replication-competent lab strains, or pseudotyped virus. It is essential to confirm experimental findings with primary cells that accurately recapitulate *in vivo* mucosal infection sites. Further understanding of the strategies HIV-1 utilizes to evade the innate response will allow for better ideas on how to increase the innate immune response to HIV-1.

The existence of a yet-undiscovered mechanism of HIV-1 restriction that is dependent on phosphorylation cannot be overlooked (63). Pretreatment MDMs with Vpx enhances the rate of HIV-1 cDNA synthesis (101), suggesting that the decrease in reverse transcription kinetics conferred by SAMHD1-mediated modulation of dNTP levels negatively regulates the rate of proviral DNA synthesis in non-dividing cells. When transcription is silenced, integrated proviral DNA can lead to latency (102). Although SAMHD1 is highly expressed in cells purported to harbor latent provirus (19, 103) and the HIV-1 proviral promoter is activated by transcription factors (104), the effect of SAMHD1 expression on latency development or reversal has not been explored. It is possible that SAMHD1 utilizes its nucleic acid binding ability to restrict HIV-1 infection postintegration, although a recent study confirmed SAMHD1 exerts no effect on HIV-1 Gag synthesis, viral particle release, and virus infectivity in 293T cells transfected with a proviral DNA construct (55). SAMHD1 may exert a direct effect on proviral DNA through binding, as purified recombinant SAMHD1 was shown to bind *in vitro* transcribed fragments of *gag* and *tat* cDNA (72), or indirect effects may occur due to SAMHD1 modulation of inflammatory pathways. It is plausible that suppression of latency reactivation by SAMHD1 would further prevent activation of the innate antiviral response. Although viral nucleic acids can be sensed by IFI16 or cGAS in the absence of SAMHD1 (24, 105), whether other pro-inflammatory pathways are affected by SAMHD1 expression remains unknown.

Discovering the mechanisms used by HIV-1 to avoid innate immune sensors is critical for the design of new therapies to eradicate HIV-1 infection. Therapeutic strategies aiming to inhibit host factors that promote HIV-1 replication and to stimulate the immune response could diminish viral infection and transmission. Current work aims to determine whether a role exists for drugs targeting SAMHD1. Expression of SAMHD1 can increase the susceptibility of HIV-1 to nucleoside reverse transcriptase inhibitors by reducing the levels of competitive dNTPs (106–109), suggesting modulation of SAMHD1 function may be a means to enhance drug effectiveness. Conversely, as SAMHD1 expression enables immune evasion by HIV-1 (13), it is tempting to hypothesize that SAMHD1 could be used as a drug target to enhance the innate immune response to viral infection. However, research is just beginning to uncover mechanisms to modify the dNTPase activity of SAMHD1 (110, 111). Importantly, as an ISG and a negative regulator of the innate immune system, SAMHD1 may be involved in an unknown negative feedback loop aimed at modulating the complex and delicate system of inflammatory pathways.

The effect of SAMHD1 on IFN-I induction during viral infection should be further studied *in vivo*. Although initial robust IFN-I responses can lead to an upregulation of antiviral genes and a block in infection, chronic immune hyperactivity could lead to desensitization of IFN-I and an eventual suppression of antiviral gene expression. This phenotype was observed when Sandler et al. manipulated the IFN-α2a response to SIV infection in rhesus macaques (112). The dismantling of the antiviral state after long-term IFN-α2a treatment led to an increase in SIV reservoir size and an accelerated CD4^+^ T-cell loss (112). Studies are necessary to determine whether stimulation of the IFN-I response through inhibition of SAMHD1 function leads to chronic inflammation and progression to AIDS *in vivo*.

## Conclusion

The identification of SAMHD1 as a regulator of the innate immune response to viral infection has led to the development of an exciting field of research. The structural and functional studies of SAMHD1 connect the physiology of HIV-1 infection to the innate antiviral response and the dynamic regulatory mechanisms in cells. Further work will aid in the development of stratagems to enhance the antiviral effects of the intrinsic immune system.

## Author Contributions

JA wrote the manuscript with input and edits from CG and LW.

## Conflict of Interest Statement

The authors declare that the research was conducted in the absence of any commercial or financial relationships that could be construed as a potential conflict of interest.
